# Implementation Mapping: Using Intervention Mapping to Develop Implementation Strategies

**DOI:** 10.3389/fpubh.2019.00158

**Published:** 2019-06-18

**Authors:** Maria E. Fernandez, Gill A. ten Hoor, Sanne van Lieshout, Serena A. Rodriguez, Rinad S. Beidas, Guy Parcel, Robert A. C. Ruiter, Christine M. Markham, Gerjo Kok

**Affiliations:** ^1^Center for Health Promotion and Prevention Research, University of Texas Health Science Center at Houston School of Public Health, Houston, TX, United States; ^2^Department of Work and Social Psychology, Maastricht University, Maastricht, Netherlands; ^3^Department of Public Health, Amsterdam UMC, University of Amsterdam, Amsterdam, Netherlands; ^4^Department of Population and Data Sciences, University of Texas Southwestern Medical Center, Dallas, TX, United States; ^5^Department of Psychiatry, University of Pennsylvania, Philadelphia, PA, United States; ^6^Department of Medical Ethics and Health Policy, University of Pennsylvania, Philadelphia, PA, United States

**Keywords:** implementation, dissemination, adoption, intervention mapping, adaptation, implementation strategies, mechanisms of change, health promotion

## Abstract

**Background:** The ultimate impact of a health innovation depends not only on its effectiveness but also on its reach in the population and the extent to which it is implemented with high levels of completeness and fidelity. Implementation science has emerged as the potential solution to the failure to translate evidence from research into effective practice and policy evident in many fields. Implementation scientists have developed many frameworks, theories and models, which describe implementation determinants, processes, or outcomes; yet, there is little guidance about how these can inform the development or selection of implementation strategies (methods or techniques used to improve adoption, implementation, sustainment, and scale-up of interventions) (1, 2). To move the implementation science field forward and to provide a practical tool to apply the knowledge in this field, we describe a systematic process for planning or selecting implementation strategies: Implementation Mapping.

**Methods:** Implementation Mapping is based on Intervention Mapping (a six-step protocol that guides the design of multi-level health promotion interventions and implementation strategies) and expands on Intervention Mapping step 5. It includes insights from both the implementation science field and Intervention Mapping. Implementation Mapping involves five tasks: (1) conduct an implementation needs assessment and identify program adopters and implementers; (2) state adoption and implementation outcomes and performance objectives, identify determinants, and create matrices of change objectives; (3) choose theoretical methods (mechanisms of change) and select or design implementation strategies; (4) produce implementation protocols and materials; and (5) evaluate implementation outcomes. The tasks are iterative with the planner circling back to previous steps throughout this process to ensure all adopters and implementers, outcomes, determinants, and objectives are addressed.

**Discussion:** Implementation Mapping provides a systematic process for developing strategies to improve the adoption, implementation, and maintenance of evidence-based interventions in real-world settings.

## Introduction

The ultimate impact of health innovations depends not only on the effectiveness of the intervention, but also on its reach in the population and the extent to which it is implemented properly. The research to practice translation process includes the development of interventions, testing their effectiveness, and ensuring they are adopted, implemented, and maintained over time. However, many research findings are never translated into policy and/or practice, or are done so very slowly, often years after its evidence has been established and with variable levels of implementation and maintenance (3). Program users do not always implement a program as it was intended, leaving out certain elements, or making alterations without careful consideration. This can compromise completeness and fidelity of implementation and subsequently program effectiveness (4, 5). Failing to appropriately implement effective interventions, guidelines, or policies severely limits the potential for patients and communities to benefit from advances in health promotion, medicine, and public health.

In the last decade, implementation science has emerged as the potential solution to this major problem (6, 7). Implementation science refers to the scientific study of methods to increase the adoption, implementation, and maintenance of evidence-based practices, programs, policies, and guidelines (6, 7). The implementation science field provides various implementation theories, frameworks, and models (8, 9). These aim to describe the process of translating research into practice, understand, or explain determinants of implementation, or to evaluate implementation (8).

Despite the rapidly increasing wealth of implementation science insights and knowledge, the majority of programs still fail to systematically plan for adoption and implementation. Instead of planning for all implementation steps from the beginning (i.e., adoption, implementation, and maintenance), the identification or development of implementation strategies typically occurs after the evidence-based intervention has already been developed or following failed implementation efforts (3, 10, 11). There seems to be a high standard for developing interventions to impact health outcomes, but less rigor and thoughtfulness in developing the implementation strategies needed to deliver the intervention. Implementation strategies are methods or techniques used to improve adoption, implementation, sustainment, and scale-up of interventions (1, 2, 12). These strategies vary in their complexity, from discrete or single component strategies (2, 7). They include both the small-scale strategies to influence specific determinants and of a implementation task, and overall packages of strategies influencing adoption, implementation, and maintenance behaviors that will ultimately determine whether a program is adopted, used, and maintained over time (3–8, 11, 13). Problems related to the development and selection of implementation strategies are evident in the literature and include: little use of theory in planning or selecting implementation strategies, lack of explicit articulation of implementation goals, limited understanding of the determinants of implementation to inform strategy development and scant descriptions of the underlying mechanisms of change that are hypothesized to cause the desired effect (14–16). For example, in a study by Davies et al. that reviewed 235 studies, authors reported that only 23% used theory to inform design of implementation strategies (14).

Nevertheless, the field has made significant strides in understanding and categorizing implementation strategies described in the published literature (7) and has suggested general approaches for selecting and describing strategies used (1, 17). These efforts have greatly advanced the field of implementation science. Still, there is little guidance on how to systematically select or plan implementation strategies at multiple ecologic levels to increase adoption, implementation, and sustainability of evidence based interventions nor how to effectively use implementation science theories and frameworks to inform the process. Thus, although useful for better understanding the types of implementation strategies that have been used, the existing inventories do little for program planners attempting to identify the most effective implementation strategies given a complex set of conditions and determinants influencing program use (1).

Researchers and practitioners alike are often forced to plan, develop, or select implementation strategies with very little information about what might work and little consideration about the mechanisms underlying potential change (18, 19). To move the implementation science field forward and to close the research-to-practice gap, a systematic process is needed to help plan for dissemination and implementation of evidence-based interventions that considers determinants, mechanisms, and strategies for effecting change. In this paper, we describe how Intervention Mapping is used to plan or select implementation strategies, a process we call *Implementation Mapping*.

## Intervention Mapping

Intervention Mapping is a protocol that guides the design of multi-level health promotion interventions and implementation strategies (13). Since its inception, a key feature of Intervention Mapping (Step 5) has been its utility for developing strategies to enhance the adoption, implementation, and maintenance of clinical guidelines (13) and evidence-based interventions (20–26).

Intervention Mapping consists of six steps: (1) conduct a needs assessment or problem analysis by identifying what, if anything, needs to be changed and for whom; (2) create matrices of change objectives by crossing performance objectives (sub-behaviors) with determinants; (3) select theory-based intervention methods that match the determinants, and translate these into strategies, or applications, that satisfy the parameters for effectiveness of the selected methods; (4) integrate the strategies into an organized program; (5) plan for adoption, implementation, and sustainability of the program in real-life contexts by identifying program users and supporters and determining what their needs are and how these should be fulfilled; (6) generate an evaluation plan to conduct effect and process evaluations to measure program effectiveness (13). Essentially, Steps 1–4 focus on the development of multilevel interventions to improve health behaviors and environmental conditions, Step 5 focuses on the development of implementation strategies to enhance program use, and Step 6 is used to plan the evaluation of both the program itself and its implementation.

Intervention Mapping can advance the field of implementation science via three distinct, yet interrelated, ways. First, the use of Intervention Mapping helps “design for dissemination” (27) a concept that means considering implementation during the development of the intervention. Intervention Mapping does so by guiding planners through a systematic process that engages stakeholders in the development of a program, policy, or practice that is likely to be both effective and usable. Second, IM can be used to systematically adapt existing evidence-based interventions to align them with new populations, geographic regions, or implementation contexts. Third, and most relevant for this paper, Intervention Mapping can help planners to develop, select, or tailor implementation strategies to increase adoption, implementation, and sustainability. Since its inception, a key feature of IM, has been its utility for developing implementation strategies to enhance the adoption, implementation, and sustainability (20–26), nevertheless, its utility has only recently been recognized by implementation scientists (12, 15, 17, 27). Thus, using Intervention Mapping for initial program development, for program adaptation, and/or for planning implementation can reduce the gap between the development of effective clinical practices and programs and their actual use in healthcare settings and communities (28).

Depending on what the evidence-based intervention is that will be implemented, a planner may choose to use all six steps of Intervention Mapping starting with Step 1, or simply Step 5. The distinction lies in whether or not there is an existing “intervention.” If, for example, the task is to develop an intervention to implement clinical practice guidelines at multiple levels of an organization (e.g., changing patient and provider behavior) and/or there are no specific products (activities, training, materials) to be implemented yet, planners should start with Step 1 of Intervention Mapping because they are developing a multi-level intervention that will, in turn, need to be implemented. If, however, there is an existing evidence-based intervention (at one or more levels) that has been developed and tested, planners can focus on how to get this intervention adopted, implemented, and maintained and begin with Intervention Mapping Step 5. Intervention Mapping Step 5 is what we refer to as *Implementation Mapping*.

## Implementation Science + Intervention Mapping = Implementation Mapping

**Figure 1:**
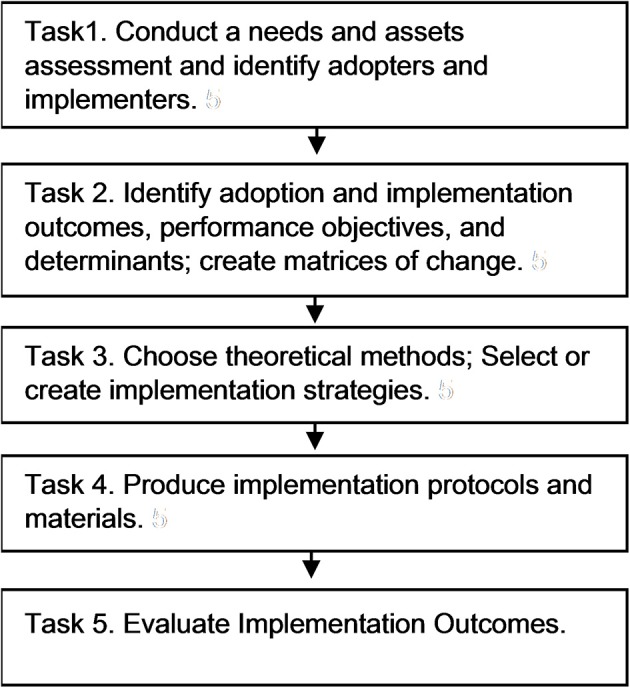
Implementation mapping process.

Implementation Mapping includes insights from both the implementation science field and from Intervention Mapping. In Implementation Mapping described here, we expand on the four tasks associated with Intervention Mapping Step 5 (identify program implementers, state outcomes and performance objectives for program use, construct matrices of change objectives, design implementation strategies) (13). Here we provide additional details for selecting and developing implementation strategies. Implementation Mapping involves five specific tasks: (1) conduct a needs assessment and identify program adopters and implementers; (2) state adoption and implementation outcomes and performance objectives, identify determinants, and create matrices of change objectives; (3) choose theoretical methods and select or design implementation strategies; (4) produce implementation protocols and materials; and (5) evaluate implementation outcomes. The five tasks are iterative with the planner circling back to previous tasks throughout to ensure all adopters and implementers, outcomes, determinants, and objectives are addressed; see Figure 1.

### Task 1. Conduct an Implementation Needs Assessment

In Implementation Mapping Task 1, planners conduct (or describe results of) a needs and assets assessment. This is sometimes referred to as identification of barriers and facilitators of implementation. Here we involve all agents including adopters, implementers, and those responsible for maintaining the evidence-based intervention in processes to identify actions needed to implement the program and determinants (barriers and facilitators) of implementation. Ideally this should have happened in Intervention Mapping step 1, but very often, a program planner has insufficient information about the implementation setting and process before the interventions has been developed.

Often, the identification and engagement of implementers occurs late in the intervention development process after the intervention is developed or proven successful and sometimes even after an implementation strategy has been selected. This can lead to low levels of implementation and maintenance because the selected strategy does not address the most salient determinants of implementation, because it does not fit well within the context, or other reasons. Therefore, the first task in Implementation Mapping is to identify program adopters and implementers. During an intervention development effort adopters and implementers may have already been a part of the team. Planners should ensure that all adopters and implementers important to implementation have been identified and are (still) involved. The questions that need to be answered at the end of task 1 are: (a) Who will decide to adopt and use the program? (b) Which stakeholders will decision makers need to consult? (c) Who will make resources available to implement the program? (d) Who will implement the program? (e) Will the program require different people to implement different components? And (f) Who will ensure that the program continues as long as it is needed (13)?

The identified stakeholders are not only stakeholders at the individual level, but also at all environmental levels. The results of the needs and assets assessment often highlight the need to target multiple adopters and implementers within an implementation setting. For example, while adopters may sometimes also be responsible for program implementation, this is not always the case. Clinic administrators may choose to adopt an evidence-based intervention to improve patient outcomes while physicians, nurses, and other staff are responsible for implementing the intervention with patients. For complex interventions, there may be different adopters and implementers for program components at different levels (clinic or school level vs. provider or teacher level). At the individual level, adopters' or implementers' attitudes toward innovations or new programs can influence decisions to adopt or implement the program. Alternatively, at the organizational level, a clinic may lack resources or personnel to implement new systems or protocols. To identify all actors and potential barriers and facilitators to implementation, the needs assessment is essential, and may require initial brainstorms within the implementation planning group and literature reviews, but also interviews with potential adopters and implementers or observations within the setting.

Wallerstein and Duran (29) describe the potential for Community-based Participatory Research (CBPR) to ensure that efforts to understand and improve implementation strategies promote reciprocal learning and incorporate community theories into these efforts. Implementation Mapping emphasizes the application of principles and processes of community based participatory research and planning and engagement of stakeholders at multiple levels. These “core processes,” described as such in the original Intervention Mapping protocol, are fundamental throughout the course of planning implementation strategies and particularly when conducting an assessment of implementation barriers, facilitators, needs, and resources. Including individuals who may adopt, implement, or use the program in understanding contextual and motivational issues and in planning and selecting implementation strategies can help address issues by ensuring integration of the local community's or clinic's priorities, perspectives, and practices (29). This also helps ensure that materials, methods, and strategies fit the local context (30–32). Additionally, creating a program in partnership with a community can help leverage community networks for implementation and dissemination (33). Thus, we encourage use of a participatory approach to implementation planning that includes potential adopters, implementers, and maintainers in implementation planning from the beginning of the planning process (34). Consistent with Diffusion of Innovation Theory, Implementation Mapping also encourages the use of a linkage system in which new change agents, program champions, and representatives of those with actual responsibility for implementation are included in the planning group (31, 35, 36). This engagement is essential to gain a realistic understanding of what organizational resources, staffing, financial, and other factors are needed for implementation (29).

Cabassa et al. (37) used Intervention Mapping combined with a community-based participatory planning approach to adapt a healthcare manager intervention focused on improving health of Hispanics with serious mental illness. They used a community advisory board with researchers and stakeholders to review the original intervention and make initial modifications. To ensure that adaptations were acceptable, they then conducted patient focus groups and stakeholder interviews. Following further adaptation based on input, they then used Intervention Mapping to develop an implementation plan, and conducted a pilot study to assess intervention feasibility, acceptability, and preliminary effectiveness. The authors highlight the differences between traditional knowledge translation approaches and a CBPR approach to implementation where stakeholders and partners participate collaboratively to understand and create strategies to improve implementation (38). They used Intervention Mapping to guide the process.

### Task 2. Identify Adoption and Implementation Outcomes, Performance Objectives, Determinants, and Change Objectives

In Implementation Mapping task 2, implementation planners state adoption and implementation outcomes and performance objectives, identify determinants, and develop matrices of change objectives. Outcomes are specific to each adopter and implementer. If adoption and implementation involve multiple actors such as administrators, physicians, and patient navigators, each may have their own adoption and implementation outcomes or performance objectives depending on their role. Performance objectives are essentially the tasks required to adopt, implement, or maintain a program. Adoption and implementation outcomes are often straightforward and simply state the key actor or actors and the adoption, implementation, or maintenance goal. Table 1 lists the adoption and implementation outcomes of the *Peace of Mind* program, an intervention to increase mammography screening among patients of community health centers. Table 1 also provides examples of outcomes from the *Long Live Love* program, a curriculum about love, relationships, and sexuality for secondary schools and vocational schools (see also https://www.langlevedeliefde.nl/docenten/english). After identifying adoption and implementation outcomes, planners state performance objectives for each outcome. Performance objectives, shown in Table 1, are the specific steps, or sub-behaviors, that adopters and implementers must perform to meet the overall adoption and implementation outcomes (13). Performance objectives make clear “*who* has to do *what*” for the program to be adopted, implemented, and continued. Performance objectives are action oriented and do not include cognitive processes such as “know” or “believe.” For adopters, the question is: “*What do [adopters] have to do in order to make the decision to use [the program]?”* These actions may, for example, include comparing the new evidence-based intervention to existing practices, gathering feedback and support from potential implementers, or signing a formal agreement to adopt.

**Table 1:** Implementation outcomes and performance objectives: select examples.

**Program: Peace of mind** **(****23**, **28****)**		
**Setting: Clinic-based**		
**Target: role**	**Adoption, Implementation, and Maintenance Outcomes**	**Performance objectives**
Clinic decision maker: Adopter	The management team at clinic decides to adopt the Peace of Mind program (PMP) as indicated by the clinic director signing a memorandum of understanding.	1. Agree to participate in PMP 2. Agree to expand mammography services 3. Agree to participate in evaluation 4. Provide a program champion 5. Gain support from stakeholders
Patient navigator: Implementer	The patient navigator will complete PMP telephone counseling with eligible patients and complete appointment reminder calls.	1. Search schedule for upcoming appointments 2. Conduct telephone barrier counseling 3. Make three attempts to reach patient via phone before appointment
Program champion: Maintainer	The program champion will ensure clinic leadership maintains PMP as part of the clinic's standard practice for every appointed mammography patient after initial funding is withdrawn.	1. Discuss with decision makers the continuation of the PMP after funding 2. Work with decision makers to continue the contractual arrangements for increased mammography services 3. Assure that mammography and no-show rates continue to be reported (and remain stable or on upward trend)
**Program: Long live love** **(****29****–****32****)**		
**Setting: School-based**		
**Target: role**	**Implementation outcomes**	**Performance objectives**
Teacher: Implementer	1. Teachers reflect and improve on their implementation behavior regarding sexual, reproductive health (SRH) lessons	1.1. Teachers reflect critically on their implementation behavior regarding SRH 1.2 Teachers self-monitor and improve the weaknesses in the implementation behavior regarding SRH
	2. Teachers deliver LLL to students completely	2.1. Teachers cover all 6 lessons of LLL (completeness = 80% of program) 2.2. Teachers us all program materials of LLL in each lesson 2.3. Teachers cover the most important components of each lesson, as indicated in the teacher manual
	3. Teachers deliver LLL to students according to the guidelines in the teacher manual (fidelity)	3.1. Teachers read the teacher manual as preparation for each lesson 3.2. Teachers deliver each LLL lesson to students according to the teacher manual
	4. Teachers deal adequately with the most common difficulties that arise during implementation of SRH	4.1. Teachers create a safe and trusted atmosphere in the classroom 4.2. Teachers teach all themes in LLL without shame or taboos interfering with the quality of the lessons 4.3. Teachers handle personal questions of students addressed to themselves depending on their personal need to answer these questions

To create performance objectives for implementers, we ask: “*What do the program implementers need to do to deliver the essential program components?* Implementation performance objectives may include attending trainings, gathering materials, or updating protocols; Table 1 contains examples of performance objectives from existing projects. And for those responsible for program continuation: “*What do they need to do to maintain the program?* Posing these questions may seem obvious, however, they help the planner articulate the exact actions required to put a health promotion intervention into use, details that are not always clear when seeking to develop or select implementation strategies. Answers to these questions are often informed by the needs assessment. Findings from the needs assessment not only help identify performance objectives but also the factors influencing whether or not these actions are carried out (determinants). In this way, Implementation Mapping tasks 1 and 2 are iterative. Through the assessment, planners may hear directly from adopters and implementers about the steps required within their setting to achieve the outcomes. Subsequently during task 2, planners may validate the performance objective with the key actors in the implementation setting.

Next, planners identify personal determinants for adopters and implementers. Determinants answer the question of “why?” Why would an implementer deliver the program as planned? (39–41). The barriers and facilitators to implementation are also determinants. Some of these determinants can also be found in the implementation science frameworks or can be theoretical constructs from health promotion theories such as the Social Cognitive Theory (39), Theory of Planned Behavior/Reasoned Action Approach (40), or the Health Belief Model (41). Essentially, determinants are modifiable factors internal to the adopters and implementers that influence their adoption and implementation behavior (13). They are the cognitive reasons why an individual would perform the desired behavioral outcome (in this case an implementation task). For example, outcome expectations, a construct from Social Cognitive Theory (also present in Theoretical Domains Framework), can influence adoption decisions. If a clinic administrator has positive outcome expectations that an evidence-based intervention will increase vaccination uptake within her clinic, she may choose to adopt the program. Alternatively, if she has negative outcome expectations or does not expect the vaccination rate in her clinic to change much due to the evidence-based intervention, she may not adopt the program. Again, Implementation Mapping task one informs this stage of the process as the determinants are often identified through the needs assessment.

Planners then create matrices of change objectives, shown in Table 2 (25). Matrices cross performance objectives with personal determinants to produce change objectives. They answer the question: *What has to change in this determinant in order to bring about the performance objective?* Change objectives are the discrete changes required in each relevant determinant that will influence achievement of the performance objective. In Table 2, the first performance objective for Peace of Mind is for the Patient Navigator to search the schedule for appointments, and the relevant determinant is awareness of the Peace of Mind program. These change objectives become the blueprint for developing (or selecting) implementation methods and strategies.

**Table 2:** Partial matrices of change objectives for selected examples.

**Program: Peace of mind** **(****23**, **28****)**			
**Behavioral outcome: Patient navigator will complete PMP telephone counseling with eligible patients and complete**			
**Appointment reminder calls**			
**Performance objectives**	**Determinants**		
	**Awareness and perceptions of PMP**	**Outcome expectations**	**Skills and Self-efficacy**
Patient Navigator searches schedule for upcoming appointments	AP.1.1. Describe requirements of the PMP intervention AP.1.2. Describe clinic data system AP.1.3. Describe protections for patient information		
Patient Navigator conducts telephone barrier counseling	AP.2.1. Describe PMP as a protocol-driven intervention AP.2.2. Describe PMP as not too complex and fairly easy to implement AP.2.3. Describe PMP as better than current practice	OE.2.1. Expect that the PMP will help women keep appointments better than current practice OE.2.2. Expect that mammography can help women detect cancer early when it is more curable OE.2.3. Expect that increasing mammography services and kept appointments will contribute to lowering mortality from breast cancer	SSE.2.1. Demonstrate skills for initiating conversation SSE.2.2. Demonstrate skills for determining women's intention for keeping appointment SSE.2.3. Demonstrate skills for eliciting barriers and using barrier scripts SSE.2.3. Demonstrate skills for supporting conversation with active listening
**Program: Long Live Love** **(****29****–****32****)**			
**Behavioral Outcome: Teachers Deal Adequately With The Most Common Difficulties That Arise During Implementation Of Srh**			
**Performance objectives**	**Determinants**		
	**Attitude**	**Self-efficacy**	**Skills**
1. The teacher integrates the theme of homosexuality as self-evident during all lessons of Long Live Love	A 1.1 Express the importance of a positive attitude of a teacher toward homosexuality during the application of the lessons	SE 1.3 Express confidence in the ability to protect students with feelings of homosexuality against a feeling of discomfort or social pressure.	S 1.4 Demonstrate how he/she protects students with homosexual feelings from a feeling of discomfort.
2. Teachers intervene on Homo-negative behavior of students	A 2.3 Express the importance of taking timely measures when students act homo-negatively in the classroom.	SE 2.2 Express confidence in ability to take measures when students act homo-negatively in the classroom.	S 2.1 Demonstrate skills to constantly being alert of homo-negative signs or behavior of students during the lessons.

### Task 3. Select Theoretical Methods and Design Implementation Strategies

In Task 3, planners choose theory- or evidence-based methods to influence the determinants identified in Task 2. They also select or design implementation strategies to operationalize those methods.

Theory-based methods include techniques to influence determinants of implementation (13). These methods can focus on either the individual level (the knowledge, attitudes, and skills of the implementer), or at the organizational level aimed at influencing organizational change directly (e.g., creating institutional commitment and strong organizational leadership). For example, a planner may need to employ information, consciousness raising, persuasive communication, and modeling (theoretical methods) to increase knowledge, address attitudes, and influence outcome expectations (determinants) among potential program adopters (13, 42). Parcel et al. (43) indicate the importance of organizational change for implementation of health promotion interventions, with school health as example. They identify a number of relevant organizational level methods for change, among others: Institutional commitment and strong organizational leadership, primarily from the superintendent, and technical assistance and resources regarding the health promotion intervention. To influence the organizational level, ten Hoor et al. (44) applied the method institutional commitment and strong leadership to the implementation of their strength-based physical exercise intervention. Regular meetings with school managements guaranteed proper participation from the schools and improvement of the study. Multiple methods may be necessary to adequately address a single determinant, and methods often influence more than one determinant. Bartholomew et al. (13) and Kok et al. (42) provide a taxonomy of theory-based methods applicable at the individual- and organizational-levels. Specific methods from the taxonomy relevant to program adoption, implementation, and maintenance include those to increase knowledge; change awareness and risk perception; change attitudes, beliefs, and outcome expectations; change social influence; increase skills, capability, and self-efficacy; change environmental conditions; change social norms and social support; and change organizations, communities, and policies (13). Table 3 provides an example of selected methods and strategies from implementation of the *Long Live Love* program. A key feature included in this table is consideration of “parameters” of methods used. Parameters represent the guidelines or conditions necessary for a particular change method to be effective. For example, for modeling to be effective, the behavior (of the model) must be reinforced. Decision makers (e.g., clinical medical directors) may not decide to implement a new program simply because a medical director (with whom they identify) has done so. They also must observe that her implementation behaviors were reinforced.

**Table 3:** Methods and applications for teachers' implementation of Long Live Love: selected examples on determinants Self-efficacy and Skills.

**Methods**	**Parameters**	**Applications/strategies**	**How population, context, and parameters were taken into account**
Behavioral journalism	Credible message; model gives reasons for adopting new behavior, and states perceived reinforcing outcomes received	Rotating photo's, role-model stories and films	Population*:* Interviews with teachers were used in several aspects of the website to realize a platform by and for teachers. Context*:* Photo's and interviews were based on a structure in which first the problem is presented as well as the experience and the relevance of this problem followed by the search for the most effective solution with a description of failures and success factors. Parameters*:* The interviewed teachers were selected to present a diverse selection in teaching experience, in geographic location and personal characteristics and were coping models, instead of mastery models, to increase the identification.
Modeling	Attention, remembrance, self-efficacy and skills, reinforcement of the model, identification with model, coping instead of mastery model, demonstrate relevant skills	Rotating photo's, role-model stories and films	Population*:* To create a platform for and by teachers, teachers were interviewed which formed the content for role-model stories and films. Photos of teachers were taken to increase reliability and credibility as well as to lure teachers to the website. Context*:* The interviews were used to fill in the main content of the website. Parameters*:* Interviewed teachers were selected on personal characteristics, on geographic location, and on experience to create a database of diverse teachers that the target group could identify with. The interviewed teachers were all coping models.

Next, planners select or design implementation strategies to operationalize methods (readers familiar with Intervention Mapping may recall that the operationalization of methods are referred to as *practical applications)*. As previously mentioned, we use the term implementation strategies in Implementation Mapping to refer to both the small-scale strategies to influence specific determinants and change objectives and to the overall package of strategies influencing adoption, implementation, and maintenance behaviors. For example, a fact sheet with heat maps outlining high risk areas is an example of a discrete strategy aimed at increasing knowledge about a health problem among potential program adopters (45). Alternatively, a face-to-face training accompanied by an instruction manual and call script is an example of a multi component implementation strategy to increase knowledge, self-efficacy, and skills for program implementers (13, 46). While the process we describe here lends itself to *developing* implementation strategies that match the determinants of implementation behavior, we can also use this method to select strategies that have been used elsewhere.

Table 4 (47) includes information from previous tasks organized into a single table by stage (adoption, implementation, or maintenance), agents, determinants, and change objectives. For example, the Peace of Mind program uses role modeling in a webinar to increase clinic decision makers' skills and self-efficacy to adopt the program.

**Table 4:** Peace of mind program implementation intervention plan.

**Stage**	**Agent**	**Determinants/change objectives**	**Theoretical change methods**	**Practical applications**
Adoption	Clinic decision maker	Awareness/perceptions of PMP Positive attitudes about the innovation – has a relative advantage, not overly complex (from CFIR construct: characteristics of the innovation) Outcome Expectations Skills and Self-efficacy Feedback and reinforcement	PMP program information Persuasion Role modeling	Email blast to BHC members with PMP informational video and link to pre-adoption survey Webinar to BHC members covering evidence-based approaches to breast cancer prevention, PMP information and adoption steps Adoption meeting held with interested clinics Financial assistance to clinic Assistance with connecting to mobile providers to increase screening (as needed)
Implementation	All	Awareness/perceptions Outcome Expectations Skills and Self-efficacy Feedback and Reinforcement	Cue to participate Communication Mobilization Organizational Consultation/Planning	Invite clinic staff to participate in stakeholder group (templates for invitation email) Email template for site visit (including requested participants) and site visit questionnaire Site visit planning meeting Program implementation guide, clinic handbook, stakeholder manual & computer assisted PMP scripts reviewed during participatory stakeholder meetings Implementation readiness checklist Stakeholder meetings to support implementation (continue after reminder calls begin). E-newsletter shared with stakeholders
Implementation	Program champion navigator	Awareness/Perceptions Outcome Expectations Skills and Self-efficacy Feedback and Reinforcement	Information Persuasion Skill building and guided practice Modeling Monitoring and feedback Technical assistance/capacity building Facilitation Vicarious reinforcement	Face to face training held over two 4 h sessions. Training was submitted to Texas for CEU certification for community health workers and social workers BHC navigators model EBI behavior and provide ongoing implementation support on-site PMP research team available via email, phone and training booster sessions as needed Paperwork processes to provide funds for patients needing financial assistance from PMP

*Adapted from Highfield (23, 28)*.

In the implementation science literature, Powell et al. (7) identified 73 implementation strategies that can be used in isolation or combination in implementation research and practice. Although comprehensive lists of implementation strategies and their definitions such as these are very important and useful, there is currently little guidance in the implementation science literature about how to select among these strategies to address determinants of implementation. Thus, in practice, the selection (or development) of strategies does not always logically follow from determinants identified. Using Task 3 in Implementation Mapping allows the planner to make decisions about strategy selection or development that logically follow the previous Implementation Mapping steps. The starting point for selection of strategies should always be their suitability to adequately address the determinants. Intervention Mapping draws upon a large body of evidence regarding which methods fit which determinants (42, 48). Additionally, it is very important that the methods are translated into a practical strategy in a way that preserves the parameters for effectiveness and fits with the target population, culture, and context (42). For example, a parameter for role modeling is that the role model needs to show coping or overcoming a barrier, rather than already mastering a skill. By adhering to the parameters of methods, strategies will be more effective for influencing implementation. To have an implementation strategy/implementation intervention to successfully implement a specific intervention/health program.

**Figure 2:**
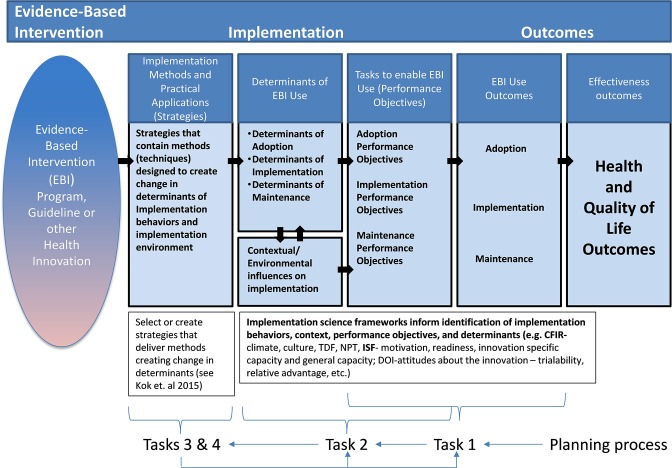
Implementation mapping logic model.

Figure 2 illustrates how implementation strategies influence health outcomes through their impact on the determinants and behaviors of those responsible for program adoption and implementation and its influence on the implementation context. Similar to logic models of the health promotion program developed using Intervention Mapping, this figure illustrates how implementation strategies can influence the determinants of implementation behaviors (detailed as performance objectives for adoption, implementation, and maintenance) which in turn influence implementation outcomes.

### Task 4. Produce Implementation Protocols and Materials

The next task in Implementation Mapping is to produce implementation protocols, activities and/or materials. Similar to Step 4 in Intervention Mapping, this requires planners to create design documents, draft content, pretest and refine content, and produce final materials. Even when selecting already existing strategies [e.g., from the Expert Recommendations for Implementing Change (ERIC) list] (7) the content within these strategies must be defined. Using Implementation Mapping, it is clear what messages, methods, and materials are needed rather than simply having selected a general strategy. Design documents are shared between planners and production teams, and they are created for each document or other materials that are a part of the implementation strategy. While no two design documents will be the same, they may include the following types of information: purpose of the material, intended audience, targeted determinants and change objectives, theoretical methods, draft content, a description of appropriate imagery, or a flowchart. For example, a planner might want to produce a testimonial video highlighting program successes in the community. This video will be posted on the program's website and target future adopters. A design document from the planners may include the following: (1) the overall purpose of the video; (2) a description of the potential adopters; (3) determinants such as knowledge, outcome expectations, and perceived social norms and associated change objectives; (4) a list of the relevant theoretical methods such as modeling, persuasive communication, and information; and (5) draft interview questions to ask the video's subject. This document provides the production team with all of the information necessary to conduct an interview and produce the testimonial video. These design documents do not only support the development of implementation interventions, but can also help evaluation and potential adaptation of implementation interventions.

### Task 5. Evaluate Implementation Outcomes

Interventions cannot be effective if they are not implemented, and their effectiveness will be compromised if they are implemented incompletely. Therefore, implementation outcomes are essential preconditions for achieving desired changes in behavior, health, or quality of life outcomes (49). Following Implementation Mapping tasks 1–4 increases the likelihood of developing implementation strategies that address identified barriers and enable implementation. Nevertheless, it is essential to evaluate whether or not these strategies have led to intended adoption, implementation, and sustainability outcomes.

Understanding implementation generates information to improve the intervention and its delivery, and for interpreting its effects on intended outcomes. Implementation evaluation and process evaluation are terms that are often used interchangeably and essentially assess the extent to which implementation strategies fit well within the context, are delivered with fidelity and are addressing identified needs (50, 51). Process and implementation evaluation can answer questions such as who the program reached, to what extent was it delivered as planned (to whom, what level of fidelity, whether theory, and evidence-based change methods were applied correctly). Because implementation is highly dependent on context, process evaluation questions can also include those that assess the organizational factors that influenced intervention adoption, use, and/or maintenance including understanding what were the barriers and facilitators to implementation.

Procter and colleagues defined several types of implementation outcomes including acceptability, adoption, appropriateness, feasibility, fidelity, implementation cost, penetration, and sustainability (49). In this task we describe how to use the preceding tasks to develop a plan to evaluate implementation and determine the impact of implementation strategies developed following tasks 1–4.

Analogous to Step 6 (Evaluation Plan) of Intervention Mapping, this task (Task 5) in Implementation Mapping helps the planner write effective process evaluation questions, develop indicators and measures for assessment, and specify the process/implementation evaluation design. Using Implementation Mapping, the planner describes expected implementation outcomes (for adoption, implementation, and/or maintenance) and performance objectives. The performance objectives delineate the specific implementation actions needed to deliver the intervention. These can be used to develop instruments to assess fidelity. Likewise the identification of determinants of implementation and creation of matrices of change objectives that state the needed changes in determinants to produce implementation outcomes, help identify important potential mediators or moderators of implementation outcomes and can again be used to develop measures to detect change in those mediators or moderators.

Following identification of process evaluation questions and measures, it is important to consider potential designs for assessing implementation outcomes. Efforts to implement evidence-based interventions are often complex, employ multilevel implementation strategies, and involve different stakeholders. The use of mixed methods approaches is particularly useful for evaluating implementation outcomes (52, 53); quantitative approaches can help confirm hypthesized relationships between implementation strategies, their impact on determinants, and the subsequent impact on implementation outcomes, while qualitative methods can explore important contextual factors influencing these relations and obtain deeper and more nuanced information about reasons for successes and failures (54). Palinkas et al. (54) provide recommendations for mixed methods approaches including the use of purposeful sampling in mixed methods implementation research.

A critical perspective in the use of Implementation Mapping for planning and evaluating implementation strategies is that, like Intervention Mapping, it is an iterative endeavor. It is unlikely, for example, for the needs and asset assessment (Task 1) to identify all barriers and facilitators to implementation and that these will likely emerge during the planning process, particularly when choosing appropriate applications of change strategies to influence determinants. Likewise, during process evaluation, it may be obvious that some key determinant was missed or that the delivery approach is not maximizing reach. The framework allows for planners to cycle back to previous tasks to more accurately reflect the mechanisms influencing implementation as well as make changes to the strategies to maximize impact.

### Implementation Logic Model

The products of Tasks 1–5 of Implementation Mapping can be presented in a model that illustrates the logic of how the strategies will affect implementation and effectiveness outcomes (see Figure 2). The logic goes from left to right with the innovation (intervention, program, policy, practice) on the far left followed by implementation strategies that deliver methods that influence determinants that change implementation behaviors and conditions and lead to implementation and ultimately effectiveness outcomes. The planning process, however, goes from right to left beginning by articulating desired outcomes and the adoption, implementation, and maintenance behaviors and conditions that will bring about those outcomes, then describing the determinants that lead to those behaviors and conditions, and finally selecting methods and developing strategies that will ultimately bring about desired outcomes. The logic model created as part of the process for planning or selecting implementation strategies helps describe the mechanisms through which we expect the implementation strategies to work. This, together with the matrices of change objectives produced in Task 2, represent blueprint or maps for the implementation strategies and guide decisions along the development or selection process. The implementation logic model is useful for both planning the implementation strategies and for designing their evaluation.

### Using Implementation Models to Inform Implementation Mapping

The Implementation Mapping process provides a framework for using implementation models for planning or selecting implementation strategies. For example, the Interactive Systems Framework (ISF) (55) can help identify key actors including adopters and implementers within particular settings. Reach, Effectiveness, Adoption, Implementation, and Maintenance, or RE-AIM, may help implementation strategy planners organize implementation outcomes at multiple levels including individual and organizational levels (56). Additionally, the Consolidated Framework for Implementation Research (CFIR) can help guide decisions about contextual factors that may influence program adoption and implementation (57). This can inform the development of performance objectives or determinants that will enter into the matrices constructed in Task 2. Combined, these models can be used to develop implementation strategies that take into account specific contexts for program adoption and implementation.

For example, they can help the planner identify program targets that go beyond effectiveness outcomes and consider adoption, implementation and maintenance (e.g., RE-AIM). In Task 1, they can be used to inform who the adopters and implementers may be and in Task 2, describe the necessary actions to adopt or deliver a program, practice, or policy.

The CFIR (57) and the Interactive Systems Framework (ISF) can help planners identify contextual and motivational factors relevant to program adoption and implementation. They can also help identify the types of capacity building that may be required to enhance implementation. The CFIR describes constructs related to implementation including (perceived) interventions characteristics (e.g., the source of the intervention), outer setting (e.g., patient needs and resources), inner setting (e.g., the implementation climate), individuals' characteristics (e.g., self-efficacy) and the implementation process (e.g., opinion leaders) (57, 58). It can therefore be useful when identifying individuals involved in implementation or in control of certain contextual factors (Task 1) and can also help identify actions needed to change implementation behaviors or contexts and their determinants (Task 2). For example, in studies implementing a Chronic Care Model (CCM) in primary care settings, implementation facilitators included a number of CFIR constructs such as engaged leadership, positive beliefs about the model, networks and communication, organizational culture, implementation climate, and structural characteristics of the setting (59). Barriers included lack of leadership engagement, lack of readiness for implementation, and poor execution (59). Therefore, researchers seeking to implement CCM in additional primary care settings and aiming to plan or select strategies could use Implementation Mapping informed by CFIR to describe performance objectives related to engaging leadership, building enthusiasm for CCM, and then identifying the determinants influencing these actions. IM would then help identify methods and plan or select strategies to address those determinants.

Another specific example of how CFIR may inform the Implementation Mapping process is as follows: if the CFIR construct leadership engagement is found to be an important predictor of implementation this can help create performance objectives (created by asking: *What does the leader have to do to increase engagement*?) as well as determinants (*Why would they engage?*). Likewise, CFIR constructs related to perceptions of the innovation (e.g., relative advantage) can point to potential determinants of both adoption and implementation behaviors. Table 4 includes CFIR informed determinants (relative advantage and complexity) and how they fit in the mapping process.

The ISF and its Readiness concepts (Readiness = Motivation × Innovation Specific Capacity × General Capacity- R = MC^2^) can help identify determinants of (Task 2) and methods (Task 3) for enhancing adopters' and implementers' readiness for implementation. Further, using the ISF, planners can think through the process of adoption and implementation at multiple levels and identify key actors at each of them (60). Another framework that is often used to understand determinants of behavior and guide implementation is the Theoretical Domains Framework (TDF) (55). The TDF includes 84 constructs listed under one of the 14 domains, all derived from the 83 theories of behavior and behavior change identified (61). It has been used to identify barriers of HPV-related clinical behaviors for general practitioners and practice nurses (62); to understand anesthesiologists' and surgeons' routine pre-operative testing behavior in low-risk patients (63); and to understand treatment adherence of adults with cystic fibrosis (64). *Note that TDF constructs can be determinants (related to the wanted behavior–such as self-efficacy) as well as methods (*e.g., *goal setting). In the systematic and iterative process of Implementation Mapping, these belong to* ***task 2****and* ***task 3****, respectively*.

Thus, informed by these frameworks and guided by the implementation mapping protocol, program planners can carefully select key implementers, articulate implementation behaviors and determinants, and then select methods and strategies to address them.

### Examples

One example of the application of Implementation Mapping is the development of strategies to implement the “Focus on Strength” program by ten Hoor et al. (65, 66). The Focus on Strength program is a school-based physical activity intervention that included 30% additional strength exercises in the physical education classes (about 15 min per session, 3 times per week) to especially reach overweight children who may be less fit but stronger than their classmates, thus allowing them to have some success and build self-efficacy. Additionally, teachers gave monthly motivational lessons to promote autonomous motivation of students to become more physically active outside school. In task 1, the planners identified adopters and implementers: managers and teachers. In task 2, they identified adoption and implementation outcomes, and their determinants. While the addition of the extra lessons seemed necessary, this was very difficult to implement in schools with already time-constrained curricula. After consulting the implementers (particularly the physical education teachers, but also the managers and planners), “time” was identified as an important potential barrier. In task 3, the planners chose methods (such as participatory problem solving and technical assistance) and strategies (teacher workshops and a workbook). To facilitate implementation, they decided (together with the implementers) to limit the extra strength component in the physical education lessons to 30% of the physical education time (about 15 min per lesson or 45 min per week) and 1 motivational lesson per month (about 10 lessons per year). This improved feasibility and facilitated adoption, implementation, and maintenance of the program. In task 4, the planning group developed and successfully used teaching protocols and materials. This way, understanding the implementation setting, including key actors (e.g., curriculum planners, directors, and teachers) and potential barriers and facilitators to adoption and implementation, potential reasons not to adopt or implement the intervention can be overcome.

Another example, described in a recently published study, also used Implementation Mapping (Intervention Mapping Step 5) to plan an implementation intervention to increase adoption, implementation and maintenance of the Peace of Mind Program, an intervention to increase mammography screening among patients of federally qualified health centers (FQHCs) (25). The authors describe how the planning group, including stakeholders, participated in brainstorming and discussing answers to questions posed by each of the Implementation Mapping tasks. They identified clinic leaders as adopters and mammography program staff and patient navigators as implementers. They then identified performance objectives and determinants based on feedback from stakeholders and using the CFIR (57, 58) “process of implementation” and “inner setting” domains to help inform the identification of both motivational and contextual factors influencing participation. This helped in the identification of performance objective and determinants. They then matched theoretical methods with determinant and operationalized them as strategies (25).

## Discussion

Despite significant advances in clinical, health promotion, and policy research that produce effective intervention, the gap between research and practice limits their impact on improving population health (3, 67). Closing this research to practice gap requires powerful strategies to address the multi-level barriers and facilitators to adoption, implementation, and maintenance needed to accelerate and improve delivery of evidence-based interventions. The study of and use of implementation strategies is central to the National Institutes of Health's (NIH's) mission of increasing the impact of the nation's investment in health-related research (68). Implementation science literature demonstrates a growing body of work on dissemination and implementation models and frameworks in the last several years (8, 9). These frameworks describe determinants, systems and processes necessary for active dissemination and implementation as well as implementation outcomes; yet, they leave some gaps in procedural knowledge on how to use these frameworks to inform the development of effective implementation strategies. As a result, few studies use theory in developing implementation strategies and sometimes researchers are not aware of the evidence-base of the methods they employ (14). This paper described a detailed systematic process for developing implementation strategies that is informed by theory, evidence, and participatory approaches to planning. Through the development of logic models, Implementation Mapping can also help better define and understand the mechanisms through which implementation strategies lead to desired outcomes.

Despite efforts to better classify implementation strategies (7) and better articulate who enacted the strategy, its influence on determinants, and its effectiveness (69), confusion remains about how to develop them and what the mechanisms of action may be. There has been much confusion in the field, for example, related to a failure to distinguish between mechanistic (theoretical methods or techniques) that cause changes in behavior, and how they are operationalized in the practice or community setting (strategy). For example, Ivers et al. (70) state that the use of audit and feedback “is based on the *belief* that healthcare professionals are prompted to modify their practice when given performance feedback showing that their clinical practice is inconsistent with a desirable target.” That is correct, but it is a method which has proven effectiveness and stems from theories such as Theories of Learning, Goal-setting Theory and Social Cognitive Theory (13, 42). Audit and feedback is indeed a frequently used *method* with a strong theoretical underpinning. It is also one of the “*strategies*” listed in the refined ERIC. However, the refined ERIC does not refer to the theoretical bases of their listed strategies, and the strategies listed are often broad recommendations (e.g., develop health education material) or guidelines. The different ways constructs, methods, strategies, etc., are classified across various compilations and frameworks gives room for confusion and misunderstanding. In this paper, we propose an organizing and conceptual framework to develop or select strategies that are specifically mapped to identified determinants of implementation and contain change methods powerful enough to address them.

An important contribution that Implementation Mapping can make to the field of implementation science literature is in filling the conceptual and practical gap between identifying implementation barriers and facilitators and developing or selecting implementation strategies. Without this type of systematic guidance for the development of implementation interventions, we will continue to struggle as a field in both the development and the selection of theory and evidence-based implementation strategies most likely to influence change.

Recently, authors have highlighted the need to articulate the causal pathways through which implementation strategies are effective (71). They suggest the need to link strategies to barriers and describe not only the desired proximal and distal outcomes but also the processes or mechanisms through which implementation strategies are effective (71). A foundational principle of Intervention Mapping and Implementation Mapping is the development of logic models (causal models) that illustrate the causal pathway between the implementation strategy, the methods it operationalizes (mechanisms), the determinants of implementation affected and the proximal and distal implementation outcomes. This includes changes in implementation behavioral and contextual factors, implementation outcomes, and the ultimate impact on health and quality of life.

Another recent article (72) suggests a process for creating a tailored implementation blueprint that includes identification of determinants of implementation. This suggestion is analogous to our Task 1 of Implementation Mapping and the selection or matching of strategies (as in Implementation Mapping Tasks 3 and 4). The importance of planning implementation strategies using a collaborative process including stakeholders at multiple levels is another central element of Implementation Mapping as described above. A recent example of one way to do this is conjoint analysis (72). We agree with recent recognition of the pressing need for processes to select and match strategies that fit implementation needs and contexts and believe that Implementation Mapping is a potential solution (17). Intervention Mapping for Planning Implementation Strategies, what we have called Implementation Mapping here, has already been employed by several authors (25, 26, 73–75) and recommended as an effective approach (17).

Implementation Mapping can advance the field of Implementation science by (1) elucidating mechanisms of change (i.e., how implementation strategies influence outcomes through change in implementation determinants) (2) better guiding the use of implementation models and frameworks during the planning process, and (3) improving the impact of implementation strategies on outcomes. The use of logic models of change that delineate the hypothesized relationships between causal factors (implementation barriers, contextual factors, behavior, and organizational change methods) and implementation outcomes can guide the development and selection of implementation strategies that will have the greatest potential impact on implementation and health outcomes.

Future directions include studies to better understand how existing implementation frameworks and models can inform the planning process. Although we believe that Implementation Mapping can help, we are only beginning to describe and demonstrate the best ways that implementation frameworks and models (and the constructs within them) can inform the development and selection of implementation strategies. Answers to questions about which tasks of Implementation Mapping are best informed by which models or elements of models is still evolving. Additionally, studies to explicitly test the use of Implementation Mapping as a planning framework for implementation strategies as compared to other methods can help provide evidence of the utility of the process.

## Conclusion

Too many evidence-based interventions are not put into practice, or are eventually implemented but with a significant delay. This compromises the potential of research findings in improving health care and health promotion efforts, and subsequently health outcomes. Implementation Mapping outlines a practical method for planning implementation strategies that will be optimally effective. Just as the systematic planning of health promotion and other interventions have greatly improved their effectiveness, the use of Implementation Mapping to plan implementation strategies will improve the appropriateness, quality, and impact of these strategies on implementation outcomes. Consequently this will lead to increased adoption, implementation, and sustainment of evidence based interventions and overall improvement in population health.

## Author Contributions

MF, GH, SvL, RB, and SR: wrote sections of the manuscript. GP, RR, CM, and GK: participated in the development of implementation mapping process, reviewed and edited the manuscript.

### Conflict of Interest Statement

The authors declare that the research was conducted in the absence of any commercial or financial relationships that could be construed as a potential conflict of interest.

## References

[1] Proctor EK Powell BJ McMillen JC. Implementation strategies: recommendations for specifying and reporting. Implement Sci. (2013) 8:139. 10.1186/1748-5908-8-139 24289295 PMC3882890

[2] Powell BJ McMillen JC Proctor EK Carpenter CR Griffey RT Bunger AC. A compilation of strategies for implementing clinical innovations in health and mental health. Med Care Res Rev. (2012) 69:123–57. 10.1177/1077558711430690 22203646 PMC3524416

[3] Morris ZS Wooding S Grant J. The answer is 17 years, what is the question: understanding time lags in translational research. J R Soc Med. (2011) 104:510–20. 10.1258/jrsm.2011.110180 22179294 PMC3241518

[4] Escoffery C Lebow-Skelley E Udelson H Böing EA Wood R Fernandez ME. A scoping study of frameworks for adapting public health evidence-based interventions. Transl Behav Med. (2018) 9:1–10. 10.1093/tbm/ibx067 29346635 PMC6305563

[5] Brownson RC Tabak RG Stamatakis KA Glanz A Implementation, dissemination, and diffusion of public health interventions. In: Glanz K, Rimer BK, Viswanath K, editors. Health Behavior: Theory, Research, and Practice 5th ed. San Francisco, CA: John Wiley & Sons (2015). p. 301–26.

[6] Eccles MP Mittman BS Welcome to implementation science. Implement Sci. (2006) 1:1 10.1186/1748-5908-1-1

[7] Powell BJ Waltz TJ Chinman MJ Damschroder LJ Smith JL Matthieu MM. A refined compilation of implementation strategies: results from the Expert Recommendations for Implementing Change (ERIC) project. Implement Sci. (2015) 10:21. 10.1186/s13012-015-0209-1 25889199 PMC4328074

[8] Nilsen P. Making sense of implementation theories, models and frameworks. Implement Sci. (2015) 10:53. 10.1186/s13012-015-0242-0 25895742 PMC4406164

[9] Tabak RG Khoong EC Chambers DA Brownson RC. Bridging research and practice: models for dissemination and implementation research. Am J Prevent Med. (2012) 43:337–50. 10.1016/j.amepre.2012.05.024 22898128 PMC3592983

[10] Glasgow RE Chambers D. Developing robust, sustainable, implementation systems using rigorous, rapid and relevant science. Clin Transl Sci. (2012) 5:48–55. 10.1111/j.1752-8062.2011.00383.x 22376257 PMC5439908

[11] Hull L Athanasiou T Russ S. Implementation science: a neglected opportunity to accelerate improvements in the safety and quality of surgical care. Ann Surg. (2017) 265:1104–12. 10.1097/SLA.0000000000002013 27735828

[12] Powell BJ Garcia KG Fernandez ME Implementation Strategies. In: Chambers D, Vinson CA, Norton WE, editors. Optimizing the Cancer Control Continuum: Advancing Implementation Research. New York, NY: Oxford University Press (2019) p. 98–120

[13] Bartholomew-Eldredge LK Markham C Ruiter RA Fernandez ME Kok G Parcel G Planning Health Promotion Programs: An Intervention Mapping Approach. 4th ed San Francisco, CA: Jossey Bass (2016).

[14] Davies P Walker AE Grimshaw JM. A systematic review of the use of theory in the design of guideline dissemination and implementation strategies and interpretation of the results of rigorous evaluations. Implement Sci. (2010) 5:14. 10.1186/1748-5908-5-14 20181130 PMC2832624

[15] Powell BJ Fernandez ME Williams NJ Aarons GA Beidas RS Lewis CC. Enhancing the impact of implementation strategies in healthcare: a research agenda. Front Public Health. (2019) 7:3. 10.3389/fpubh.2019.00003 30723713 PMC6350272

[16] Waltz TJ Powell B Fernandez ME Abadie B Damschroder L. Choosing implementation strategies to address contextual barriers: diversity in recommendations and future directions. Implement Sci. (2019) 14:42. 10.1186/s13012-019-0892-4 31036028 PMC6489173

[17] Powell BJ Beidas RS Lewis CC Aarons GA McMillen JC Proctor EK. Methods to improve the selection and tailoring of implementation strategies. J Behav Health Serv Res. (2017) 44:177–94. 10.1007/s11414-015-9475-6 26289563 PMC4761530

[18] Benjamin Wolk C Powell BJ Beidas RS Contextual influences and strategies for dissemination and implementation in mental health. (2015 2015–09-10). In: Oxford Handbooks Online. Oxford University Press. Available online at: http://www.oxfordhandbooks.com/view/10.1093/oxfordhb/9780199935291.001.0001/oxfordhb-9780199935291-e-12

[19] Powell BJ Proctor EK Learning from implementation as usual in children's mental health. Implement Sci. (2016) 11:26–7. 10.1186/1748-5908-8-92 26923462 PMC4770686

[20] Bartholomew LK Cushman WC Cutler JA Davis BR Dawson G Einhorn PT. Getting clinical trial results into practice: design, implementation, and process evaluation of the ALLHAT Dissemination Project. Clin Trials. (2009) 6:329–43. 10.1177/1740774509338234 19587068 PMC2897824

[21] Cabassa LJ Gomes AP Meyreles Q Capitelli L Younge R Dragatsi D. Using the collaborative intervention planning framework to adapt a health-care manager intervention to a new population and provider group to improve the health of people with serious mental illness. Implement Sci. (2014) 9:178. 10.1186/s13012-014-0178-9 25433494 PMC4255430

[22] Donaldson A Lloyd DG Gabbe BJ Cook J Finch CF. We have the programme, what next? Planning the implementation of an injury prevention programme. Injury Preven. (2017) 23:273–80. 10.1136/injuryprev-2015-041737 26787739 PMC5537515

[23] Flash CA Frost ELT Giordano TP Amico KR Cully JA Markham CM. HIV Pre-exposure prophylaxis program implementation using intervention mapping. Am J Prevent Med. (2018) 54:519–29. 10.1016/j.amepre.2017.12.015 29433956

[24] Forbes LJ Forster AS Dodd RH Tucker L Laming R Sellars S. Promoting early presentation of breast cancer in older women: implementing an evidence-based intervention in routine clinical practice. J Cancer Epidemiol. (2012) 2012:835167. 10.1155/2012/835167 23213334 PMC3505655

[25] Highfield L Valerio M Fernandez ME Bartholomew-Eldridge K. Development of an implementation intervention using intervention mapping to increase mammography among low income women. Front Public Health. (2018) 6:300. 10.3389/fpubh.2018.00300 30416992 PMC6212476

[26] Peskin MF Hernandez BF Gabay EK Cuccaro P Li DH Ratliff E. Using intervention mapping for program design and production of iCHAMPSS: an online decision support system to increase adoption, implementation, and maintenance of evidence-based sexual health programs. Front Public Health. (2017) 5:203. 10.3389/fpubh.2017.00203 28848729 PMC5554483

[27] Brownson RC Jacobs JA Tabak RG Hoehner CM Stamatakis KA. Designing for dissemination among public health researchers: findings from a national survey in the United States. Am J Public Health. (2013) 103:1693–9. 10.2105/AJPH.2012.301165 23865659 PMC3966680

[28] Klesges LM Estabrooks PA Dzewaltowski DA Bull SS Glasgow RE. Beginning with the application in mind: designing and planning health behavior change interventions to enhance dissemination. Ann Behav Med. (2005) 29:66–75. 10.1207/s15324796abm2902s_10 15921491

[29] Wallerstein N Duran B. Community-based participatory research contributions to intervention research: the intersection of science and practice to improve health equity. Am J Public Health. (2010) 100:S40–6. 10.2105/AJPH.2009.184036 20147663 PMC2837458

[30] Coughlin SS Smith SA Fernández ME Handbook of Community-Based Participatory Research. New York, NY: Oxford University Press, Incorporated (2017).

[31] Bartholomew LK Fernandez M James S Leerlooijer J Markham C Reinders J Using Intervention Mapping to Adapt Evidence-Based Programs to New Settings and Populations. In: Bartholomew LK, Parcel GS, Kok G, Gottlieb NH, Fernandez ME, editors. San Francisco, CA: Jossey-Bass (2011).

[32] Fernandez MEB LK Vidrine JI Reininger B Kransy S Wetter DW The Role of Translational Research in Behavioral Science and Public Health. Hackensack, NJ: World Scientific (2014).

[33] Glasgow RE Marcus AC Bull SS Wilson KM. Disseminating effective cancer screening interventions. Cancer. (2004) 101(5 Suppl):1239–50. 10.1002/cncr.20509 15316911

[34] Bartholomew Eldredge LK Markham CM Kok G Ruiter RA Parcel GS Intervention Mapping Step 5: Program Implementation Plan. San Francisco, CA: Jossey-Bass (2016).

[35] Havelock RG The utilisation of educational research and development. Br J Educ Technol. (1971) 2:84–98. 10.1111/j.1467-8535.1971.tb00552.x

[36] Orlandi MA. The diffusion and adoption of worksite health promotion innovations: an analysis of barriers. Prevent Med. (1986) 15:522–36. 10.1016/0091-7435(86)90028-9 3774782

[37] Cabassa LJ Druss B Wang Y Lewis-Fernández R. Collaborative planning approach to inform the implementation of a healthcare manager intervention for hispanics with serious mental illness: a study protocol. Implementat Sci. (2011) 6:80. 10.1186/1748-5908-6-80 21791070 PMC3169485

[38] Parikh P Simon EP Fei K Looker H Goytia C Horowitz CR. Results of a pilot diabetes prevention intervention in East Harlem, New York City: Project HEED. Am J Public Health. (2010) 100(Suppl 1):S232–9. 10.2105/AJPH.2009.170910 20147680 PMC2837455

[39] Bandura A. Health promotion by social cognitive means. Health Educ Behav. (2004) 31:143–64. 10.1177/1090198104263660 15090118

[40] Ajzen I. The theory of planned behaviour: reactions and reflections. Psychol Health. (2011) 26:1113–27. 10.1080/08870446.2011.613995 21929476

[41] Rosenstock IM Strecher VJ Becker MH. Social learning theory and the health belief model. Health Educ Quart. (1988) 15:175–83. 10.1177/109019818801500203 3378902

[42] Kok G Gottlieb NH Peters GJ Mullen PD Parcel GS Ruiter RA. A taxonomy of behaviour change methods: an Intervention Mapping approach. Health Psychol Rev. (2016) 10:297–312. 10.1080/17437199.2015.1077155 26262912 PMC4975080

[43] Parcel GS Simons-Morton BG Kolbe LJ. Health promotion: integrating organizational change and student learning strategies. Health Educ Quart. (1988) 15:435–50. 10.1177/109019818801500405 3230018

[44] Ten Hoor GA Plasqui G Ruiter RAC Kremers SPJ Rutten GM Schols AMWJ. A new direction in psychology and health: Resistance exercise training for obese children and adolescents. Psychol Health. (2016) 31:1–8. 10.1080/08870446.2015.1070158 26155905 PMC4662099

[45] Peskin MF Hernandez BF Markham C Johnson K Tyrrell S Addy RC Sexual health education from the perspective of school staff: implications for adoption and implementation of effective programs in middle school. J Appl Res Child. (2011) 2:9.

[46] Highfield L Rajan SS Valerio MA Walton G Fernandez ME Bartholomew LK. A non-randomized controlled stepped wedge trial to evaluate the effectiveness of a multi-level mammography intervention in improving appointment adherence in underserved women. Implement Sci. (2015) 10:143. 10.1186/s13012-015-0334-x 26464110 PMC4604615

[47] Highfield L Hartman MA Mullen PD Rodriguez SA Fernandez ME Bartholomew LK. Intervention mapping to adapt evidence-based interventions for use in practice: increasing mammography among African American Women. Biomed Res Int. (2015) 2015:160103. 10.1155/2015/160103 26587531 PMC4637430

[48] Koutoukidis DA Lopes S Atkins L Croker H Knobf MT Lanceley A. Use of intervention mapping to adapt a health behavior change intervention for endometrial cancer survivors: the shape-up following cancer treatment program. BMC Public Health. (2018) 18:415. 10.1186/s12889-018-5329-5 29587699 PMC5869761

[49] Proctor E Silmere H Raghavan R Hovmand P Aarons G Bunger A. Outcomes for implementation research: conceptual distinctions, measurement challenges, and research agenda. Administr Policy Mental Health Serv Res. (2011) 38:65–76. 10.1007/s10488-010-0319-7 20957426 PMC3068522

[50] Century J Rudnick M Freeman C A framework for measuring fidelity of implementation: a foundation for shared language and accumulation of knowledge. Am J Eval. (2010) 31:199–218. 10.1177/1098214010366173

[51] Harachi TW Abbott RD Catalano RF Haggerty KP Fleming CB. Opening the black box: using process evaluation measures to assess implementation and theory building. Am J Commun Psychol. (1999) 27:711–31. 10.1023/A:1022194005511 10676545

[52] Palinkas LA Aarons GA Horwitz S Chamberlain P Hurlburt M Landsverk J. Mixed method designs in implementation research. Adm Policy Ment Health. (2011) 38:44–53. 10.1007/s10488-010-0314-z 20967495 PMC3025112

[53] Proctor EK Landsverk J Aarons G Chambers D Glisson C Mittman B. Implementation research in mental health services: an emerging science with conceptual, methodological, and training challenges. Adm Policy MentHealth. (2009) 36:24–34. 10.1007/s10488-008-0197-4 19104929 PMC3808121

[54] Palinkas LA Horwitz SM Green CA Wisdom JP Duan N Hoagwood K. Purposeful sampling for qualitative data collection and analysis in mixed method implementation research. Admin Pol Ment Health. (2015) 42:533. 10.1007/s10488-013-0528-y 24193818 PMC4012002

[55] Wandersman A Duffy J Flaspohler P Noonan R Lubell K Stillman L. Bridging the gap between prevention research and practice: the interactive systems framework for dissemination and implementation. Am J Commun Psychol. (2008) 41:171–81. 10.1007/s10464-008-9174-z 18302018

[56] Glasgow RE McKay HG Piette JD Reynolds KD. The RE-AIM framework for evaluating interventions: what can it tell us about approaches to chronic illness management? Patient Educ Counsel. (2001) 44:119–27. 10.1016/S0738-3991(00)00186-5 11479052

[57] Damschroder L Aron D Keith R Kirsh S Alexander J Lowery J. Fostering implementation of health services research findings into practice: a consolidated framework for advancing implementation science. Implement Sci. (2009) 4:50. 10.1186/1748-5908-4-50 19664226 PMC2736161

[58] CFIR Research Team-Center for Clinical Management Research The Consolidated Framework for Implementation Research [cited 2018 December 20]. Available online at: https://cfirguide.org/

[59] Kadu MK Stolee P. Facilitators and barriers of implementing the chronic care model in primary care: a systematic review. BMC Family Pract. (2015) 16:12. 10.1186/s12875-014-0219-0 25655401 PMC4340610

[60] Atkins L Francis J Islam R O'Connor D Patey A Ivers N. A guide to using the Theoretical Domains Framework of behaviour change to investigate implementation problems. Implement Sci. (2017) 12:77. 10.1186/s13012-017-0605-9 28637486 PMC5480145

[61] Davis R Campbell R Hildon Z Hobbs L Michie S. Theories of behaviour and behaviour change across the social and behavioural sciences: a scoping review. Health Psychol Rev. (2015) 9:323–44. 10.1080/17437199.2014.941722 25104107 PMC4566873

[62] McSherry LA Dombrowski SU Francis JJ Murphy J Martin CM O'Leary JJ. ‘It's a can of worms': understanding primary care practitioners' behaviours in relation to HPV using the theoretical domains framework. Implement Sci. (2012) 7:73. 10.1186/1748-5908-7-73 22862968 PMC3523072

[63] Patey AM Islam R Francis JJ Bryson GL Grimshaw JM. Anesthesiologists' and surgeons' perceptions about routine pre-operative testing in low-risk patients: application of the Theoretical Domains Framework (TDF) to identify factors that influence physicians' decisions to order pre-operative tests. Implement Sci. (2012) 7:52. 10.1186/1748-5908-7-52 22682612 PMC3522997

[64] Arden MA Drabble SJ O'Cathain A Hutchings M Wildman M WS16.1 ACtiF study: understanding adherence to nebuliser treatment in adults with cystic fibrosis using the Theoretical Domains Framework. J Cystic Fibros. (2016) 15:S26 10.1016/S1569-1993(16)30151-5

[65] ten Hoor GA Plasqui G Schols AMWJ Kok G. Development, implementation, and evaluation of an interdisciplinary theory- and evidence-based intervention to prevent childhood obesity: theoretical and methodological lessons learned. Front Public Health. (2017) 5:352. 10.3389/fpubh.2017.00352 29312922 PMC5743937

[66] Ten Hoor GA Rutten GM Van Breukelen GJP Kok G Ruiter RAC Meijer K. Strength exercises during physical education classes in secondary schools improve body composition: a cluster randomized controlled trial. Int J Behav Nutri Phys Activity. (2018) 15:92. 10.1186/s12966-018-0727-8 30253776 PMC6156874

[67] Hanney SR Castle-Clarke S Grant J Guthrie S Henshall C Mestre-Ferrandiz J. How long does biomedical research take? Studying the time taken between biomedical and health research and its translation into products, policy, and practice. Health Research Policy Syst. (2015) 13:1. 10.1186/1478-4505-13-1 25552353 PMC4297458

[68] National Cancer Institute National Institutes of Health Implementation Science: About IS. (2015). Available online at: https://cancercontrol.cancer.gov/IS/about.html

[69] Leeman J Birken SA Powell BJ Rohweder C Shea CM. Beyond “implementation strategies”: classifying the full range of strategies used in implementation science and practice. Implement Sci. (2017) 12:125. 10.1186/s13012-017-0657-x 29100551 PMC5670723

[70] Ivers N Jamtvedt G Flottorp SA Young JM Odgaard-Jensen J French SD Audit and feedback: effects on professional practice and healthcare outcomes. Cochr Database Syst Rev. (2012) 6:CD000259 10.1002/14651858.CD000259.pub3 PMC11338587 22696318

[71] Lewis CC Klasnja P Powell BJ Lyon AR Tuzzio L Jones S. From classification to causality: advancing understanding of mechanisms of change in implementation science. Front Public Health. (2018) 6:136. 10.3389/fpubh.2018.00136 29868544 PMC5949843

[72] Lewis CC Scott K Marriott BR. A methodology for generating a tailored implementation blueprint: an exemplar from a youth residential setting. Implement Sci. (2018) 13:68. 10.1186/s13012-018-0761-6 29769096 PMC5956960

[73] Gabbe B Donaldson A Poulos R Lloyd D Cook J Finch C Planning for implementation: use of intervention mapping (Step 5) in two sports safety intervention case studies. Injury Preven. (2012) 18(Suppl 1):A58–A9. 10.1136/injuryprev-2012-040580e.23

[74] Kobel S Wartha O Wirt T Dreyhaupt J Lämmle C Friedemann E-M. Design, implementation, and study protocol of a kindergarten-based health promotion intervention. Biomed Res Int. (2017) 2017:9. 10.1155/2017/4347675 28303253 PMC5338306

[75] Hurley DA Murphy LC Hayes D Hall AM Toomey E McDonough SM. Using intervention mapping to develop a theory-driven, group-based complex intervention to support self-management of osteoarthritis and low back pain (SOLAS). Implement Sci. (2016) 11:56. 10.1186/s13012-016-0418-2 27113575 PMC4845501

